# Evaluation of Left Ventricular Diastolic Function by Echocardiography with Tissue Doppler in Systemic Sclerosis

**DOI:** 10.5935/abc.20170145

**Published:** 2017-11

**Authors:** Marina Carneiro de Freitas Roque, Percival D. Sampaio-Barros, Ana Lucia Arruda, Sergio Barros-Gomes, Derly Becker, José Lazaro de Andrade, Ana Clara Tude Rodrigues

**Affiliations:** Faculdade de Medicina da Universidade de São Paulo, São Paulo, SP - Brazil

**Keywords:** Heart Ventricles / function, Echocardiography, Doppler, Sleroderma, Systemic, Ventricular Dysfunction, Left

## Abstract

**Background:**

Systemic sclerosis (SS) is a connective tissue abnormality characterized by fibrosis of the skin and internal organs. Cardiac involvement with consequent myocardial dysfunction in SS is associated with increased morbidity and mortality.

**Objective:**

To investigate the left ventricular (LV) diastolic function in patients with SS and preserved systolic function.

**Methods:**

Patients with SS were evaluated with two-dimensional echocardiography with tissue Doppler for analysis of chamber diameters, LV mass index (LVMI), indexed left atrial volume (iLAV), systolic function of both ventricles, and presence and degree of diastolic dysfunction (DD).

**Results:**

We evaluated 50 patients, divided according to the presence of DD into Group 1 (n = 25; normal diastolic function, E/A ratio ≥ 0.8, deceleration time [DT] > 150 ms and < 200 ms, and septal e’ > 8 cm/s) and Group 2 (n = 25; with DD, subdivided into type I DD [E/A < 0.8, DT > 200 ms], type II [E/A ≥ 0.8, septal e’ < 8 cm/s, iLAV > 34 mL/m^2^], and type III [E/A > 2, DT < 150 ms, septal e’ < 8 cm/s]). Type I DD was the most frequent (34%), followed by type II DD (16%). LVMI and iLAV were similar in both groups, but septal and lateral e’ were reduced only in Group 2. In Group 2, we observed that patients with moderate DD had longer disease duration (p = 0.02).

**Conclusion:**

The prevalence of type I DD was elevated in SS and associated with aging. Disease duration emerged as an important factor in moderate DD.

## Introduction

Systemic sclerosis (SS) is a diffuse connective tissue disease characterized by skin and internal organ fibrosis and thickening, vascular alterations, and eventual ischemic ulcers and visceral abnormalities.^1^ The prevalence of SS varies between 7 and 489 individuals per million persons and may vary by gender (more common in women), age (usually emerges between the third and fifth decades of life), and ethnicity (more common in the US and Australia than in Japan or Europe).^2^ SS can be clinically subdivided into a limited form of the disease, which only affects the skin of the face, hands, and feet, or a diffuse form, which occurs with thickening of the extremities and abdomen, trunk, and roots of the limbs. The cardiac involvement in SS can be primary (myositis, heart failure, cardiac fibrosis, coronary artery disease, conduction abnormalities, and pericardial disease) or secondary (resulting mainly from pulmonary fibrosis and renal insufficiency).^1-4^ Systolic and/or diastolic dysfunction (DD) may be secondary to myocardial fibrosis, left ventricular (LV) hypertrophy, hypertension, renal disease, or respiratory sleep disorder. Echocardiography reveals myocardial disease in 50% to 70% of the cases, but in most patients, cardiac dysfunction is clinically silent until the disease reaches a more advanced stage.^3-6^ Techniques derived from two-dimensional echocardiography, such as tissue Doppler, have been used for the evaluation and early detection of ventricular dysfunction in various situations.^5-7^ Their use in the study of SS, however, has been limited to studies of small size^8^ or with inadequate methodologic definition.^9^

The objective of this study was to evaluate the LV diastolic function by echocardiography associated with tissue Doppler in patients with SS.

## Method

The study included patients with SS attending the Rheumatology Outpatient Clinic of the *Hospital das Clínicas* at the Medical School of *Universidade de São Paulo* in the period between November 2010 and December 2011, of both sexes, and older than 18 years. All patients fulfilled the criteria of the American College of Rheumatology (ACR)^10^ and, subsequently, the new classification criteria of the ACR and the European League Against Rheumatism (EULAR).^11^ We included outpatients with SS without severe visceral manifestations (especially interstitial fibrosis, pulmonary hypertension, cardiomyopathy, or scleroderma renal crisis), as well as without severe comorbidities. All patients had Raynaud's phenomenon. The study was approved by the Research Ethics Committee of the *Hospital das Clínicas* and all patients signed an informed consent for participation.

### Transthoracic echocardiography

All patients underwent an initial two-dimensional echocardiography with tissue Doppler imaging (Artida, Toshiba, Japan) for a complete evaluation of the ventricular structure and function, with emphasis on an analysis of the diastolic LV function. Based on the guidelines of the American Society of Echocardiography,^12^ we obtained measurements of the systolic and diastolic diameters of the LV with the two-dimensional mode to calculate the ejection fraction (Teichholz method). We also obtained the diameters of the aortic root and left atrium from the parasternal long axis view. The calculation of the LV mass was performed using the measurements of the diastolic LV thickness and cavity by the Devereux method^12^ and indexed by body surface area. The indexed left atrial volume (iLAV) was obtained from the apical two-chamber and four-chamber views by the modified Simpson method. Valvular alterations were evaluated with the two-dimensional mode, conventional Doppler, and color mapping, with the pulmonary pressure measurement obtained by tricuspid regurgitation and added to the estimate of the right atrial pressure from inferior vena cava.

### Diastolic function analysis

Transmitral Doppler measurements were obtained with the Doppler sample volume positioned on the edge of the leaflets in the apical four-chamber view to obtain the E (initial) and A (late) waves, E/A ratio, and E-wave deceleration time (DT). We also obtained tissue Doppler tracings from the apical four-chamber view with the Doppler sample volume positioned in the basal region of the septum and in the lateral mitral annulus ring for analysis of s’, e’, and a’ wave velocity.

The analysis of the diastolic function was performed based on the classification below, following the recommendations of the American Society of Echocardiography:^13^

- Normal Function: iLAV < 34 mL/m^2^, E/A 0.8-1.5, E-wave DT > 150 ms and < 200 ms, septal e' wave ≥ 8 cm/s.
- DD type I (mild): E/A < 0.8, E-wave DT > 200 ms, septal e' wave < 8 cm/s.
- DD type II (moderate): iLAV ≥ 34 mL/m^2^, E/A 0.8-1.5, E-wave DT between 150-200 ms, septal e' wave < 8 cm/s with an E/e' ratio > 13.
- DD type III (severe): iLAV > 34 mL/m^2^, E/A > 2, E-wave DT < 150 ms, septal e' wave < 8 cm/s with an E/e' ratio > 13.

Based on the diastolic function analysis, the patients were divided into two groups: Group 1, with normal diastolic function and Group 2, with DD. The patients were also analyzed in relation to the degree of DD presented. The presence of an inadequate acoustic window and decreased LV ejection fraction (< 50%) were considered exclusion criteria.

### Statistical analysis

The Kolmogorov-Smirnov test was used to assess normality. For continuous variables with normal distribution, the data are presented as mean ± standard deviation and for variables without normal distribution, as median and interquartile range (IQR). The categorical variables are presented as absolute numbers and percentages. The groups were compared using two-tailed unpaired Student's *t* test for variables with normal distribution and Wilcoxon test for variables without normal distribution. To assess the degree of DD, we used analysis of variance (ANOVA) and, subsequently, Dunnett’s test.

The statistical analyses were performed using the software JMP, version 8 (SAS Institute, Cary, NC, USA). P values < 0.05 were considered statistically significant.

## Results

We analyzed 54 patients with SS, three of whom were excluded due to inadequate acoustic window and one due to decreased LV ejection fraction, totaling 50 patients. Most (n = 46) participants were female, and their mean age was 52 ± 11 years. The median disease duration was 9 years (IQR 5-15 years). The minimum and maximum disease durations were 3 and 45 years, respectively. Only three patients had hypertension, and none had diabetes or a history of manifestation of coronary artery disease (Table 1). The echocardiographic data including LV ejection fraction, diameters of the cardiac chambers, pulmonary artery systolic pressure, ventricular mass index, and iLAV were within normal values for this group of patients (Table 2).

**Table 1:** Patients’ clinical data and medications

Variable	
Age (years)	52 ± 11
Female sex	46 (92%)
Disease duration (years) (interquartile range)	9 (5 - 15)
Depression (n, %)	3 (6%)
Hypertension (n, %)	3 (6%)
Pulmonary hypertension (n, %)	6 (12%)
Calcium channel blocker (n, %)	28 (56%)

**Table 2:** Echocardiographic data of the total sample of patients and subgroups with and without diastolic dysfunction

Variables	Total n = 50	Without DD n = 25	With DD n = 25	p
Aortic root (mm)	29 ± 3	29 ± 3	29 ± 3	0.631
Left atrium (mm)	36 ± 5	36 ± 4.7	3.7 ± 5.0	0.489
iLAV (cm/m^2^)	27 ± 8	24 ± 4.8	29 ± 10	0.417
LV diastolic diameter (mm)	44 ± 5	43 ± 4	44 ± 6	0.06
SW (mm)	9.7 ± 1.2	9.5 ± 1.1	9.9 ± 1.3	0.247
PW (mm)	29 ± 4	9.5 ± 1.1	9.9 ± 1.3	0.768
LV ejection fraction (%)	62 ± 7	63 ± 4	62 ± 3	0.172
LV mass index (g/m^2^)	88 ± 28	87 ± 20	90 ± 34	0.950
PASP (mmHg)	30 ± 14	25 ± 7	35 ± 17	0.03
E (cm/s)	84 ± 19	88 ± 17	80 ± 22	0.137
A (cm/s)	79 ± 22	68 ± 13	91 ± 23	0.0001
E/A	1.1 ± 0.3	1.3 ± 0.27	0.9 ± 0.24	0.0001
DT (ms)	188 ± 44	162 ± 25	214 ± 45	< 0.0001
Septal e' (cm/s)	8.5 ± 2.1	10.0 ± 1.6	7.1 ± 1.2	< 0.0001
Lateral e' (cm/s)	11.6 ± 2.7	13.4 ± 2.3	9.9 ± 1.9	< 0.0001
Septal E/e'	10.4 ± 3.6	8.9 ± 2.3	11.9 ± 4.1	0.005
Lateral E/e'	7.7 ± 2.7	8.3 ± 3.3	7.1 ± 1.9	0.189

DD: diastolic dysfunction; iLAV: indexed left atrial volume; LV: left ventricle; SW: septal wall; PW: posterior wall; PASP: pulmonary artery systolic pressure; E: early diastolic filling wave; A: late diastolic filling wave; DT: E-wave deceleration time; e’: tissue Doppler early diastolic wave. Unpaired Student’s t test for comparison between subgroups with and without diastolic dysfunction.

**Figure 1:**
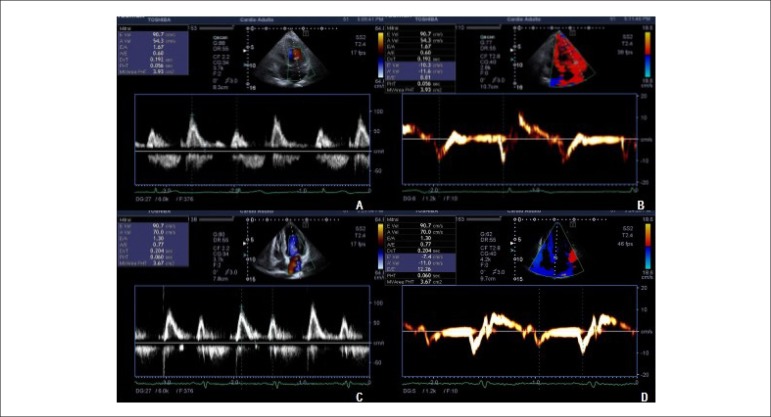
A and B: Pulsed Dopper images showing transmitral flow and septal tissue Doppler showing a normal diastolic pattern. C and D: Tissue Doppler images showing a reduced e’ wave (7.4 cm/s), compatible with moderate diastolic dysfunction (pseudonormal pattern).

The patients presented a very high prevalence of DD, with half of the study sample (25 patients) presenting some degree of DD, of whom the majority (17 patients, 34%) had mild DD (type I) and a lower portion had type II DD (8 patients, 16%), as shown in Figure 1. None of the patients had type III DD.

The groups with and without DD did not differ regarding gender or presence of hypertension. On the other hand, the group with DD was significantly older than the one without DD (58 ± 9 years *versus* 46 ± 10 years, respectively, p < 0,05). The disease duration showed no significant difference between the groups, and the median disease duration was 8 years (IQR 3.5-11.0 years) for patients without DD and 11 years (IQR 6.0-16.5 years) for those with DD (p = 0.178; Wilcoxon test). The LV mass index and the iLAV were also similar in both groups. However, the lateral and septal e' waves were reduced only in patients with DD, resulting in an increased septal E/e' ratio. The lateral E/e' relation did not differ between the groups. The pulmonary pressure was also higher in Group 2 (Table 2).

**Figure 2:**
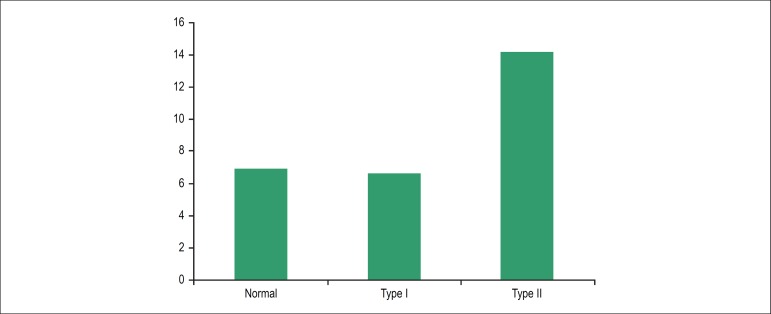
Relationship between the degree of diastolic dysfunction and disease duration (p = 0.02). Relationship between the degree of diastolic dysfunction and disease duration in years (p = 0,02)

When we analyzed the subgroups according to the degree of DD, we observed that it was not only age that was related to the presence of DD (Figure 2); for patients with moderate DD (type II), we observed that the duration of the disease was significantly prolonged when compared with patients without DD or with mild DD(p = 0,02). Patients without DD and those with mild DD had disease durations of 9 ± 7 years and 9 ± 4 years, respectively, compared with 18 ± 14 years among patients with moderate DD.

## Discussion

Cardiac involvement often occurs in SS and is usually associated with a more reserved prognosis, representing the second most common cause of death after pulmonary involvement.^14^ In order to study the presence of early cardiac involvement in SS, the present study assessed the frequency of DD in a group of patients with preserved systolic function. It has been observed that the use of Doppler tissue imaging associated with conventional echocardiography increases the diagnostic accuracy of this method.^6^ This was confirmed in our study, which observed the occurrence of DD in half of the patients studied, although this population exhibited preserved systolic function and a low prevalence of other risk factors for DD, such as hypertension, ventricular hypertrophy, or diabetes. Population studies evaluating diastolic function in community-dwelling individuals have observed that DD is mainly associated with increasing age, but is rarely found in the absence of risk factors for diastolic abnormalities, being uncommon even in elderly individuals.^15^ Similarly, Kuznetsova et al., in a study evaluating diastolic function in the general population,^16^ have shown that in subjects aged between 50-59 years (a similar age range as our cohort), the proportion of total DD reached 42%, and type I DD (abnormal relaxation) was found in about 32% and type II DD in approximately 10% of the population. These values were not very different from those found in our sample of patients with SS; the prevalence of DD was 50%, with 34% of the individuals presenting type I DD, with a prevalence possibly slightly higher for type II DD (16% of the patients with SS). This population study found, in a similar way, an association between DD with age and body mass index, in addition to the presence of comorbidities, such as high blood pressure and increased serum creatinine. It is important to emphasize that in the referred study, almost 70% of the patients with type I DD had hypertension, with this proportion increasing to almost 80% in the subgroup with type II DD. On the other hand, only three patients in our sample had hypertension, and the absence of other comorbidities strongly suggests that DD was associated with SS. It has been reported more recently a higher prevalence of DD in patients with SS when compared with individuals of the same age in the general population. Meune et al. reported the presence of mild DD in 50% of the patients with type I DD (abnormal relaxation) found in a control group.^6^ A study conducted by Hinchcliff et al. observed alteration of relaxation in only 23% of the patients with SS, and its presence was associated with increased mortality.^9^ We also observed that the degree of DD was mild in most patients, with few cases of moderate DD and no patient with severe DD. These findings suggest that the disease itself evolves more frequently with subtle diastolic changes, prevailing alterations in ventricular relaxation with intracardiac pressures still within normal limits, as demonstrated by the normal E/e’ ratio in most cases.

DD may be the result of a primary myocardial involvement in SS^16^ or may be secondary to hypertension, LV hypertrophy, pericardial diseases, and coronary disease.^17^ In our group of patients, the number of hypertensive patients or patients with other comorbidities associated with DD was not significant, which leads us to believe that the probable cause of this abnormality was the primary cardiac involvement. The presence of a LV mass index within normal values in our sample also corroborates the hypothesis that the evolution of the disease itself is the cause of the cardiac dysfunction, thus rejecting the LV hypertrophy as the cause of DD. Primary myocardial structural changes (fibrosis) may, in turn, cause DD. Tzelepis et al., using cardiac magnetic resonance imaging,^18^ have shown the occurrence of delayed enhancement (compatible with fibrosis) in about 60% of the patients with SS, even in those without evident systolic dysfunction. The presence of DD is clinically important in the evolution of patients with SS, since cardiac involvement in SS is associated with increased mortality.^9,19^ In addition, the increased LV diastolic pressures are transmitted to the lungs, with consequent pulmonary hypertension. During exercise, with the increased diastolic pressures, the pulmonary pressure may be even higher, as demonstrated in patients with SS during exercise echocardiography,^20^ leading to symptom worsening. It is important to note, however, that the absence of DD during examination does not exclude the presence of myocardial fibrosis.

When we analyzed the factors associated with DD, we observed that the patients were quite similar in regards to clinical characteristics, except in relation to the greater prevalence of DD in older individuals. Several changes in cardiac structure and function derive from the aging process, including a reduced number and increased size of myocytes, with resulting increase in connective tissue.^21,22^ In addition, dead myocytes are replaced by collagen, with consequent interstitial fibrosis, making the heart more rigid and less complacent, therefore affecting the diastolic relaxation. When we compared patients with and without DD, we observed that there was no influence of disease duration on DD. However, when we analyzed the subgroups, we observed that in addition to age, there was a significant association between disease duration and moderate DD. The association between myocardial fibrosis and longer disease duration may be related to a greater number of flares of cardiac Raynaud's phenomenon in patients with SS of long duration, since the successive vascular ischemic changes contribute to increased fibrosis. However, none of the patients, even with a longer disease course, presented important DD (restrictive pattern). It is possible that this pattern of more advanced DD may ultimately be found in patients with associated LV systolic dysfunction, but these cases were not evaluated in the present study.

Echocardiographic analysis of the cardiac function is simple and effective, allowing monitoring of the disease and early diagnosis of changes such as DD through the use of conventional Doppler associated with tissue Doppler. As the cardiac involvement is associated with a more severe progression, the demonstration of this involvement by echocardiography could indicate a need for stricter monitoring of this population, in addition to being used as an indication of therapeutic improvement.

As a limitation of this study, the relationship between DD and myocardial fibrosis can only be accepted as hypothetical since the patients did not undergo magnetic resonance imaging to test this association. The study had a cross-sectional design and we still lack long-term prognostic data, which could be associated with the presence of DD in this group of patients.

## Conclusion

DD is frequent in patients with SS and normal systolic function. It is characteristically mild and associated with more advanced age. A longer disease duration is associated with a more pronounced DD pattern.
